# *Bacillus cereus* Subacute Native Valve Infective Endocarditis and Its Multiple Complications

**DOI:** 10.1155/2020/8826956

**Published:** 2020-06-23

**Authors:** Jemimah Nallarajah, M. I. Mujahieth

**Affiliations:** Department of Medicine, Colombo South Teaching Hospital, Sri Lanka

## Abstract

*Bacillus cereus* causing infective endocarditis (IE) in a native valve is an extremely rare event, but it is reported mostly in intravenous drug abusers and other risk factors as immunosuppression, malignancy, and valvular heart disease including prosthetic heart valves. We report a case of *B.cereus* native mitral valve infective endocarditis in a 58-year-old Sri Lankan male who is not a drug abuser who presented with painless hematuria with reduced urine output. During hospital stay, he developed frequent episodes of brief focal seizures. He had undergone multiple investigations that revealed splenic abscesses, cerebral vasculitis, and glomerular nephritis with positive rheumatoid factor, cytoplasmic antineutrophil cytoplasmic antibody (C-ANCA), and cryoglobulin. The appropriate antibiotic was the prime therapeutic intervention which carried an excellent prognosis. This case highlights an unusual organism in the blood culture that caused IE warranting thorough physical examination and investigations.

## 1. Introduction

Infective endocarditis (IE) is a life-threatening condition that typically affects a person with congenital heart disease or rheumatic valvular disease or prosthetic heart valves, but there are risk factors as central venous catheter, pace maker, implantable cardioverter-defibrillator (ICD), immunosuppressive state, and malignancy which also play a role in IE. The most commonly identified causative organisms are *Staphylococcus* and *Streptococcus* (1). *Bacillus cereus* is known for its association with acute gastroenteritis and is a rare causative organism of native valve IE, but that has been reported in IVDA and in the presence of other risk factors mentioned above (2). IE causes various vascular and immunological manifestations leading to life-threatening complications. Early identification and appropriate interventions save lives.

## 2. Case Presentation

A 58-year-old male presented with intermittent low-grade fever and painless hematuria of one-month duration. On further inquiry, he had a significant weight loss of 6 kg in the period of the same duration. He was on long-term medication for diabetes mellitus without any micro- or macrovascular complications, and his glycemic control was satisfactory with oral hypoglycemic drugs. He was a school principal and not an alcoholic or smoker and denied usage of any recreational drugs.

On 10^th^ day of hospital admission, he developed three episodes of left-sided focal seizures without loss of consciousness, and each episode lasted for about two minutes.

On preliminary examination, he was afebrile, has thin built with body mass index (BMI) of 19.3 kg/m^2^, and severely pale with finger clubbing. There were no other peripheral stigmata of infective endocarditis or lymphadenopathy identified. The pulse rate was irregularly irregular with a rate of 70 beats per minute and a pansystolic murmur best heard at the apex which radiated to the axilla; apart from that, other systemic examinations were unremarkable.

**Figure 1:**
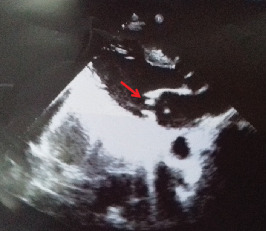
Transthoracic echocardiography; tiny 0.8 × 0.4 cm^2^ vegetation attached to the anterior mitral leaflet (arrow).

**Figure 2:**
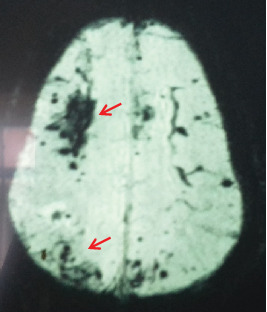
MRI/SWI image of the brain. There is a significant blooming with multiple peripheral and central bleeding foci in the cerebral hemispheres (arrow).

**Figure 3:**
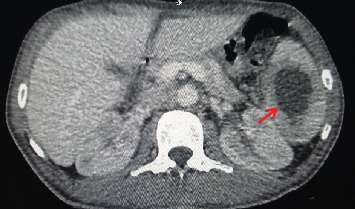
CECT abdomen; irregular hypodense ring-enhancing lesion in the parenchyma of the spleen (arrow).

He had to undergo several (Tables 1 and 2 and Figures 1–3) investigations for his diagnostic workup.

The transthoracic echocardiography demonstrated mitral regurgitation with tiny vegetation attached to the anterior mitral leaflet which was 0.8 × 0.4 cm^2^ in size (Figure 1 (arrow)). Electrocardiography (ECG) showed multiple ventricular extra systoles without atrial fibrillation.

The magnetic resonance imaging (MRI) of the brain revealed multiple foci of hemorrhage in the bilateral cerebral hemispheres and cerebellum that are suggestive of cerebral vasculitis, and there were no space-occupying lesions or cerebral abscess. Eye screening was negative for vasculitis or endophthalmitis.

The contrast-enhanced computed tomography (CECT) of the chest and abdomen showed two splenic abscesses on the parenchyma and the lateral wall (Figure 3 (arrow)); apart from that, it was unremarkable.

He developed oliguria with progressively rising creatinine and had to undergo hemodialysis twice with blood transfusions. A renal biopsy was performed that demonstrated focal segmental glomerulosclerosis (FSGS) with acute and chronic tubular interstitial nephritis. No immunosuppressive therapy had been used for rapidly progressive renal failure, but the patient recovered with antibiotic therapy.

Intravenous (IV) ciprofloxacin had been administrated for 8 weeks which was sensitive for *Bacillus cereus* that has been identified by an automated identification and susceptibility testing system (BD Phoenix). He had a protracted and prolonged hospital stay for about two months. However, he made a complete recovery. There are no relapses for two years of clinic follow-up.

## 3. Discussion

*Bacillus cereus* is an aerobic spore barer and a facultative anaerobe found in the environment and certain types of food and also can be found in the gut flora which can be isolated from the human feces. When the spores overcome the immunity of the body, they become infective and can cause systemic infections such as abscess of various sites, endophthalmitis, pneumonia, and osteomyelitis (3, 4). *Bacillus cereus* causing IE commonly affects males with the age group of 41–60 years. The mitral valve is more frequently affected among the valves followed by aortic and tricuspid (common in IVDA) valves. Mortality is higher in patients with a prosthetic heart valve than native valve (5).

In our patient, the portal of entry of this organism is not clear, but we speculate that the infection was acquired from a meal that contained the organism or *Bacillus cereus* bacteremia from the gut flora. However, he had diabetes mellitus as a risk factor which is a known cause for immunosuppression by reduced phagocytosis and impaired leukocyte chemotaxis that may contribute to the pathogenesis of IE. Diabetes with poor glycemic control carries poor prognosis. Our patient has been managed with insulin and had a satisfactory control during the ward stay.

The appropriate antibiotic therapy is very vital in the management of IE, and it should be given for at least six weeks*. B.cereus* is commonly sensitive to aminoglycosides, vancomycin, ciprofloxacin, linezolid, or clindamycin and showed resistance to beta-lactamase and cephalosporin (5).

Acute kidney injury (AKI) presented as hematuria in our case as a complication of IE. He had been offered hemodialysis for the rapidly progressive nature of renal disease and also continued the antibiotics without any immunosuppressant, and the ultimate outcome was excellent. We strongly emphasize here that cardiac auscultation is very important in the cases of hematuria to avoid misdiagnosis. In our case, especially the presence of ANCA, rheumatoid factor and cryoglobulin associated with hematuria definitely direct to a diagnosis of small vessel vasculitis, and the management with immunosuppressant could further exacerbate the infection that leads to an adverse outcome. IE is a mimicker of vasculitis. Immunological phenomena of IE may result in a positivity of cryoglobulin, ANCA, and rheumatoid factor that will become negative once the infection is completely treated (6).

Most cases of glomerulonephritis associated with subacute IE are presented as hematuria with AKI. Glomerulonephritis causing hematuria is almost always painless, and rapidly progressive glomerulonephritis (RPGN) is the most common anticipated complication or a presenting manifestation of glomerulonephritis. There are several mechanisms that have been identified in IE which cause renal manifestations: immune complex deposition, septic emboli-related infarction and abscess, antibiotic-related interstitial nephritis, and thrombotic microangiopathy. FSGS is rarely found in the association of IE which has been reported in the literature that presents as proteinuria, hematuria, and hypertension. In our case, apart from hypertension, other features were found. FSGS is the result of immune complex deposition in the glomerular basement membrane that usually is associated with hypocomplimentemia. In our patient, the compliments were normal that could be due to the antibiotic therapy prior to renal biopsy (7, 8).

A splenic abscess is a rare and a fatal complication of IE (9). Our patient did not have any symptoms or signs related to the abscess apart from low-grade fever, but it has been found incidentally on the imaging. Percutaneous aspiration had been attempted however which did not yield an adequate sample for culture. The patient had been managed effectively with antibiotics, and follow-up images showed complete resolution.

The common neurological complications are mainly due to septic emboli and present as seizure, stroke, aneurysm, and cerebral abscess (10, 11). Our patient had developed cerebral vasculitis; it is an uncommon and rare manifestation and causes microbleeds that are commonly found in the brain parenchyma or in the subarachnoid space (10). Cerebral vasculitis can be primary or secondary that needs further evaluation to exclude the differential diagnoses. A brain biopsy is the gold standard investigation to make the diagnosis of cerebral vasculitis (10). However, in the presence of IE, we did not perform it, and the patient also showed a marked clinical improvement with antibiotics only without a need of any corticosteroid pulses. Neurological complications are commonly life-threatening if left untreated. *Staphylococcus aureus* causing IE is commonly identified with neurological complications (12).

The guideline of the American Association for Thoracic Surgery has mentioned several indications and timing of surgical interventions which are considered in patients who have signs of heart failure, persistent sepsis despite adequate antibiotic therapy for more than five to seven days, severe valve dysfunction, large mobile vegetation, recurrent systemic embolization, paravalvular abscess, or cardiac fistula and the presence of neurological complications (recurrent strokes, cerebral mycotic aneurysm) (13). Even though our patient developed a neurological complication (vasculitis), it was managed successfully with antibiotics.

## 4. Conclusion

IE must be considered in any atypical presentation of a critically ill patient, and it needs to be ruled out. *Bacillus cereus* is an uncommon organism to cause native valve IE, and it warrants thorough investigations if identified in blood cultures. Timely diagnosis matters with regard to appropriate therapeutic intervention.

## Figures and Tables

**Table 1:** Summary of basic investigations.

	Normal range	1^st^ day	20^th^ day	40^th^ day	60^th^ day
White cell (×10^9^)	4-11	9.34	23.5	14.8	5.82
Neutrophils (×10^9^)	1.5–8.0	6.2	21.6	9.3	2.46
Hemoglobin (g/dL)	13.5–16.5	6.9	7.8	9.8	10.1
Platelet (×10^9^/L)	150-450	206	305	228	196
Sodium (mmol/L)	135-145	136	133	137	138
Potassium (mmol/L)	3.5-5.0	3.9	3.8	3.6	3.8
Creatinine (*μ*mol/L)	50-120	457	524	362	103
CRP (mg/L)	<6	129	194	128	16
ESR (mm/1^st^ hour)	<20	68	110	92	32
Protein (g/L)	66-83	59.2	57.2	58.8	60.3
Albumin (g/L)	35-50	22.6	24.7	25.1	31.3
Globulin (g/L)		36.6	32.5	33.7	29.0
Total bilirubin (*μ*mol/L)	5-17	11	12.3	10	6.2
ALP (IU/L)	40-140	181	155	121	92.1
ALT (U/L)	10-40	21.9	20.3	16.7	11.8
AST (U/L)	10-40	48.5	40.1	29.8	18.6
Corrected Ca (mmol/L)	2.2–2.6	2.33	2.31	2.30	2.36
Phosphate (mg/dL)	3.4–4.5	5.3	3.3	1.0	1.2
Magnesium (mmol/L)	0.85–1.1	1.0	0.99	0.89	0.9
Urine full report		Field full red cells Protein 3+	Field full red cells Protein +	58–45 Protein-nil	Nil
Urine protein creatinine ratio (>3.5 nephrotic range)		2.61			

**Table 2:** Other investigations.

Blood culture–2 sets	*Bacillus cereus*
Urine culture	No growth
Cryoglobulin	Positive
C-ANCA	Positive
P-ANCA	Negative
IgG (mg/dL) (569-1919)	1290
IgM (mg/dL) (47-147)	152
IgA (mg/dL) (61-330)	156
C_3_ (mg/dL) (55-120)	119
C_4_ (mg/dL) (20-50)	26
Hepatitis B surface antigen	Negative
Hepatitis C antibody	Negative
Cytomegalovirus IgM/IgG	Negative
Epstein-Barr virus IgM/IgG	Negative
Retroviral study	Negative
VDRL	Negative
Typhus Weil-Felix	Negative
Brucella antibody	Negative
Urine Bence Jones protein	Negative
Serum protein electrophoresis	No monoclonal bands
Antinuclear antibody (ANA)	Negative
Urine dysmorphic red cells	7%
Endoscopic studies of GIT	Unremarkable

## Data Availability

All the data supporting our case report is contained within the manuscript.

## Abbreviations

AKI: Acute kidney injury
ALP: Alkaline phosphatase
ALT: Alanine transaminase
AST: Aspartate transaminase
BMI: Body mass index
C-ANCA: Cytoplasmic antineutrophil cytoplasmic antibody
CECT: Contrast-enhanced computed tomography
CRP: C-reactive protein
ESR: Erythrocyte sedimentation rate
FSGS: Focal segmental glomerulosclerosis
GIT: Gastrointestinal tract
IE: Infective endocarditis
IV: Intravenous
IVDA: Intravenous drug abuser
MRI: Magnetic resonance imaging
SWI: Susceptibility weighted imaging
VDRL: Venereal disease research laboratory.
